# Generic sensor platform based on electro-responsive molecularly imprinted polymer nanoparticles (e-NanoMIPs)

**DOI:** 10.1038/s41378-020-00193-3

**Published:** 2020-10-19

**Authors:** A. Garcia-Cruz, O. S. Ahmad, K. Alanazi, E. Piletska, S. A. Piletsky

**Affiliations:** School of Chemistry, University of Leicester, Leicester, UK

**Keywords:** Bionanoelectronics, Nanoparticles

## Abstract

The present research describes the design of robust electrochemical sensors based on electro-responsive molecularly imprinted polymer nanoparticles (e-MIPs). The e-MIPs, tagged with a redox probe, combine both recognition and reporting functions. This system replaces enzyme-mediator pairs used in traditional biosensors. The analyte recognition process relies on the generic actuation phenomenon when the polymer conformation of e-MIPs is changing in response to the presence of the template analyte. The analyte concentration is measured using voltammetric methods. In an exemplification of this technology, electrochemical sensors were developed for the determination of concentrations of trypsin, glucose, paracetamol, C4-homoserine lactone, and THC. The present technology allows for the possibility of producing generic, inexpensive, and robust disposable sensors for clinical, environmental, and forensic applications.

## Biosensors: generic sensors using electroactive molecularly imprinted polymer nanoparticles

Robust generic electrochemical sensors have been designed based on electroactive molecularly imprinted polymer nanoparticles (e-MIPs), in which molecular imprinting (creating template-shaped recognition sites in polymers) is applied to nanoparticles. Commercial biosensor technology has made remarkable advances over the past decade, but glucose biosensors still account for the overwhelming majority of the world biosensor market. However, a team headed by Alvaro Garcia-Cruz and Sergey Piletsky at the University of Leicester, United Kingdom has succeeded in designing electrochemical sensors employing e-MIPs. The e-MIPs combine both recognition and reporting functions in a system that replaces the enzyme-mediator pairs used with conventional biosensors. Ease of production and cost efficiency are major technological advantages of this technology over traditional sensors. The authors believe that their system has considerable potential for producing generic, inexpensive, and robust disposable sensors for clinical, environmental, and forensic applications.

## Introduction

Commercial biosensor technology has rapidly increased in development over the past decade, with the global biosensor market valued at 18.6 billion USD in 2018^1^. However, despite the many technological advances in research and development, as well as the introduction of many different products, glucose biosensors still account for ~71% of the current world market for biosensors. There are many explanations, both scientific and market-related, as to why this is the case^2^. Focusing on the scientific side of this phenomenon, it is possible to conclude that the reasons why the number of commercial applications of biosensors remains limited are linked to the following:

- Poor stability of enzymes and antibodies in practical applications;
- Lack of suitable enzymes for practically important substrates and analytes; and
- Lack of an appropriate and generic approach for the transduction of binding events into a detectable electrical signal (particularly in the case of antibody-based sensors).

In an ideal world, recognition components used in biosensors would be robust, inexpensive, sensitive to all targets, capable of generating a sensor response, and suitable for integration with inexpensive and robust transducers. Unfortunately, neither enzymes nor antibodies are capable of meeting these expectations. Over the last few years, we have made several attempts to address these issues by developing robust sensors based on molecularly imprinted polymers (MIPs)^3–8^.

Molecular imprinting is the process of template-induced formation of specific recognition sites in a polymer where a template directs the positioning and orientation of the polymer functional groups by a self-assembling mechanism^2,9^. MIPs possess a unique combination of properties, such as high affinity, specificity, robustness and low price, which makes them an attractive alternative to natural receptors, enzymes and antibodies used in biosensors^10^. Recently, we developed a novel protocol for producing pseudo-monoclonal MIP nanoparticles (nanoMIPs) in the presence of an immobilized analyte that serves as a template in solid-phase synthesis^11–13^. After polymerization, high-affinity nanoparticles are extracted from the solid phase, leaving behind an immobilized analyte that can be re-used for the preparation of a new batch of polymers^14^. The process for the preparation of highly selective nanoMIPs using solid-phase synthesis is suitable for scale-up and automation^15^. NanoMIPs prepared on a solid phase are very stable, have long operational and shelf lives, can theoretically be made for any target, and can be easily functionalized with reporter moieties.

Different strategies can be applied to integrate nanoMIPs with electrochemical sensors. For example, nanoMIPs were entrapped in electro-conducting polymers for voltammetric determination of ephedrine^16^, in a PVC matrix for potentiometric measurements of cocaine and atrazine^6,17^, in nafion for voltammetric detection of vancomycin^18^ and in cross-linked electrodes using self-assembled monolayers of alkane thiols for voltammetric and impedimetric detection of 4-ethylphenol^19,20^. Here, we present a further step, important for the development of MIP sensors—the design of electroactive nanoMIPs with a reporting function.

In biosensors, redox markers are used to facilitate electron transport between enzyme catalytic sites and electrodes and in this way transform biorecognition and catalytic effects into a detectable electric signal^21^. Redox markers have low oxidation and reduction potentials, and their use helps minimize interference from unwanted electroactive species. Soluble redox markers were used in combination with MIPs for *indirect* detection of effects related to polymer–template interactions. Thus, the binding of the template to the imprinted polymer reduces the permeability of redox markers such as ferricyanide to electrodes coated with MIPs, and this can be used to measure the concentration of the analyte^22,23^. This phenomenon, called the “gate effect”, is frequently used in sensors and is a result of morphological changes in the polymer triggered by specific interactions of the polymeric layer with the template molecule^24–27^.

**Fig. 1:**
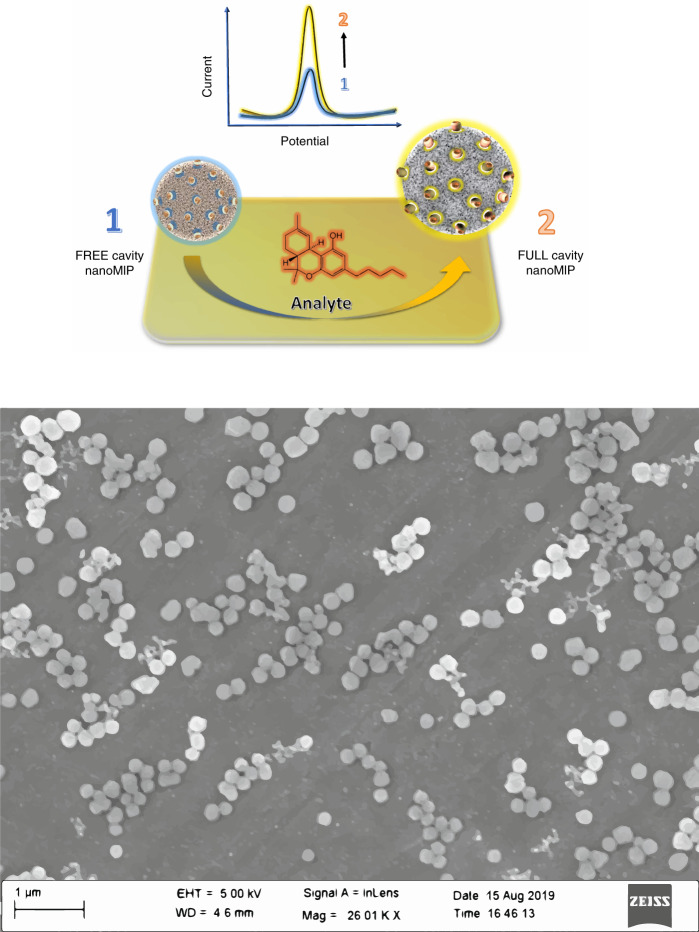
Sensor principle and microscopy analysis. **a** Schematic representation of the e-MIP response to the analyte; the analyte recognition triggered a detectable change in the polymer conformation. **b** SEM images of e-nanoMIPs for paracetamol detection

Conveniently, instead of using soluble mediators, template-responsive nanoMIPs can be covalently functionalized with redox labels using polymerizable ferrocene derivatives^28^. Ferrocene derivatives and their redox polymers have shown excellent properties, such as redox stability at low potentials, pH independence, and fast electron transfer, making them perfect as mediators and reporters^29^. In redox-labeled MIP nanoparticles (e-MIPs), the recognition of the analyte in specific binding sites would be intimately integrated with a reporting mechanism that is attenuated by conformational changes in the polymer^30^. Swelling (or shrinking) would affect the density of ferrocene moieties exposed on the surface of nanoparticles that are anchored onto the electrode. As a result, the binding event will be translated into a detectable signal that can be monitored via electrochemical techniques (Fig. 1).

**Fig. 2:**
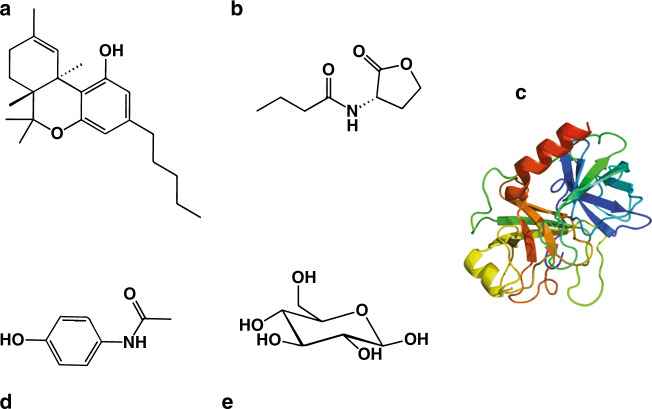
Target molecules. **a** THC, **b** C4-HSL, **c** trypsin, **d** paracetamol, and **e** glucose

The present work describes the use of this generic approach in the preparation of robust electrochemical sensors for clinical, environmental and forensic analysis. As a proof of concept, we developed sensors for the detection of trypsin, glucose, paracetamol, C4-homoserine lactone (C4-HSL), and THC (Fig. 2).

## Results and discussion

To design nanoMIPs with integrated recognition and reporting functions, we followed previously developed protocols^31^. The nanoMIP fabrication involves controlled polymerization of the monomer mixture in the presence of template molecules immobilized onto a solid phase support, typically glass beads^6^. The only exception here was the synthesis of e-MIPs imprinted with glucose, which was performed in a solution containing soluble glucose molecules. This change in the synthetic protocol was introduced due to the different requirements of the affinity of nanoMIPs for glucose, which should be at a millimolar level. The standard composition used in MIP preparation was modified through the addition of a polymerizable ferrocene derivative (see Protocols and Methods). After synthesis, high-affinity e-nanoMIPs were extracted from the solid surface by elution at an elevated temperature. The sensor fabrication used in our work relied on well-established carbodiimide coupling of e-MIP deposition onto a gold surface of screen-printed gold electrode (SPGE). coated with self-assembled monolayers of alkanethiol^32^. The general preparation protocol is shown in Table 1. The new sensors for different targets can be produced using the same protocol by replacing the template during MIP synthesis.

**Table 1:** Generic protocol for preparation of nanoMIP-based sensors

Step	Procedure
1. Solid-phase activation	Glass beads are activated in basic media and then silanized
2. Template immobilization	The analyte is covalently attached to silanized glass beads through carbodiimide amidation
3. NanoMIP synthesis on a solid phase	*(For small molecules) polymerization in organic phase*: controlled photo-polymerization
	*(For biomolecules) polymerization in aqueous phase*: controlled radical polymerization
4. Purification	Separation through thermal solid phase extraction and ultrafiltration
5. Electrode modification	*SPGE* modification with cysteamine
6. NanoMIP immobilization	Covalent attachment using carbodiimide amidation

*SPGE* screen-printed gold electrode

The size of the e-MIPs was measured by scanning electron microscopy and dynamic light scattering (DLS). A typical image of e-MIPs with diameter ∼250 nm is shown in Fig. 1. In solution, e-MIPs swell depending on the type of solvent, pH, and ionic strength. The fact that nanoMIP conformation is affected by their interaction with the template was crucial for the purposes of the present work. Previously, the swelling of MIP membranes was used for the detection of target analytes^9,33^. However, the sensor response of bulk polymers was very slow, requiring up to an hour, and fabrication protocols for such sensors were incompatible with the requirements for mass production of sensor devices. In this respect, using e-MIPs containing ferrocene in sensors would offer advantages such as a fast response, an absence of signal interference from the change in diffusion parameters of the soluble mediator, and variations in oxygen concentration. It is also much easier to integrate soluble e-MIPs with electrodes using the same protocols as in enzyme biosensors.

The e-MIP conformational changes triggered by the analyte were evidenced by measuring the diameter of nanoparticles in water using DLS. For example, the diameter of nanoMIPs imprinted with THC showed a size increase of 23% in THC solutions. Presumably, NanoMIP actuation is specific to THC and no changes were observed in presence of other molecules. Similar effects were observed for MIPs made for other targets (Table 2). These results demonstrate that actuation of the sensor response by changing the nanoMIP conformation is template specific.

**Table 2:** DLS measurements for e-nanoMIPs in solution and loaded with the analyte

Nanoparticle type	Molecule in solution	Size (nm)	PDI	Size change (%)
Specific for glucose	Water	201.8 ± 16.4	0.373	–
	Glucose	238.1 ± 8.7	0.325	18
Specific for C4-HSL	Water	186.5 ± 8.6	0.316	–
	C4-HSL	208.9 ± 4.5	0.283	12
Specific for paracetamol	Water	106 ± 20	0.312	–
	Paracetamol	128.3 ± 14	0.274	21
Specific for THC	Water	307.3 ± 9.8	0.323	–
	THC	379.2 ± 6.4	0.295	23
Specific for trypsin	Water	443.8 ± 4.8	0.196	–
	Trypsin	519.2 ± 4.4	0.134	17

*PDI* polydispersity index

The e-MIPs were covalently immobilized onto gold surfaces of screen-printed electrodes as described below. The sensor response was measured using differential pulse voltammetry (DVP) at ∼0.22 V vs Ag/AgCl, which corresponds to the oxidation peak of ferrocene.

**Fig. 3:**
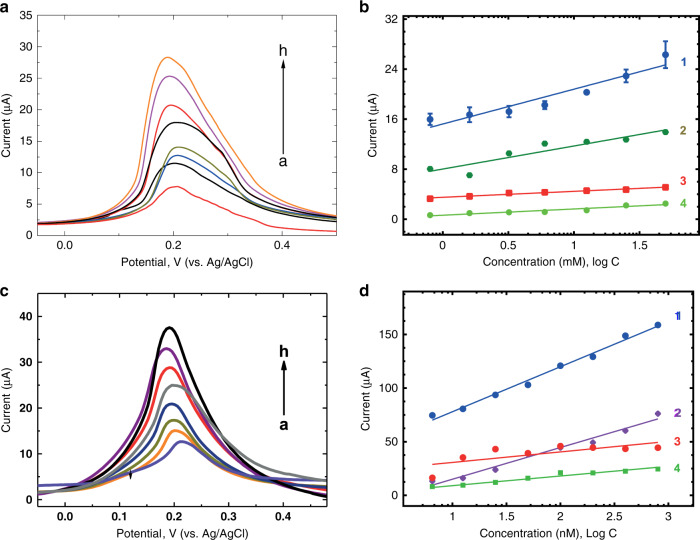
Glucose and C4-HSL sensor response and selectivity. **a** Sensor response (DPV) of glucose e-MIPs to glucose; **b** Sensor response of glucose e-MIPs to (1) glucose (2) fructose (3) maltose and (4) lactose in a concentration range of 0.8–50 mM; **c** Sensor response (DPV) of C4-HSL e-MIPs to C4-HSL; **d** Sensor response of C4-HSL e-MIPs to (1) C4-HSL, (2) C6-HSL, (3) GBL, and (4) 3-oxo-C6-HSL in a concentration range 6.25–800 nM. All experiments were tested in PBS

**Fig. 4:**
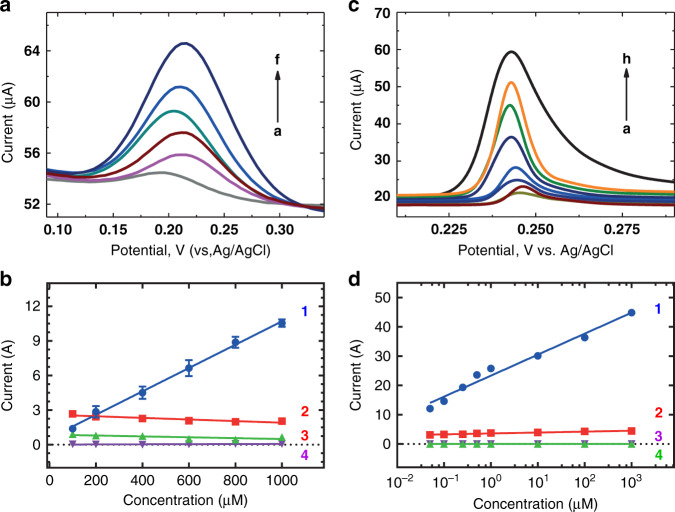
Paracetamol and THC sensor response and selectivity. **a** Sensor response (DPV) of paracetamol e-MIPs to paracetamol; **b** Sensor response paracetamol e-MIPs to (1) paracetamol, (2) caffeine, (3) procainamide, and (4) ethyl 4-aminobenzoate in a concentration range 100–1000 µM. **c** Sensor response (DPV) of THC e-MIPs to THC; **d** Sensor response THC e-MIPs to (1) THC, (2) CBDV, (3) THC-COOH and (4) caffeine in a concentration range 0.1–1000 µM. All experiments were conducted in spiked plasma

**Fig. 5:**
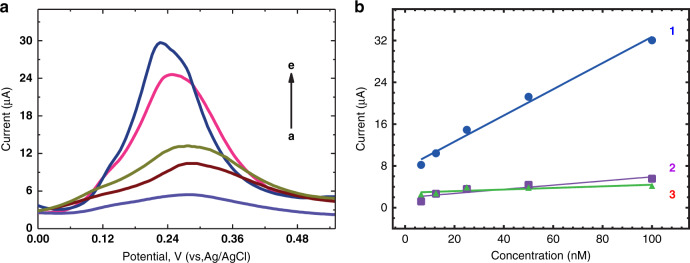
Trypsin sensor response and selectivity. **a** Sensor response (DPV) of trypsin e-MIPs to trypsin; **b** Sensor response of trypsin e-MIPs to (1) trypsin, (2) avidin and (3) pepsin in a concentration range 6.5–100 nM. All experiments were conducted in spiked plasma

To demonstrate the generic nature of the proposed approach, electrochemical sensors were developed for several targets: (a) glucose, (b) C4-HSL, (c) paracetamol, (d) THC, and (e) trypsin. In all experiments performed here, the current response of e-MIPs increased proportionally to the analyte concentration (Figs. 3–5). The sensor response was very quick, allowing for the detection of analytes within 7 min after the addition of the sample. The reason for this stems from the small dimensions of the nanoparticles, allowing for fast diffusion of analyte molecules to binding sites located on their surface.

The glucose sensor response revealed a sensitivity of 5.57 ± 0.79 µA/mM (*R*^2^ = 0.97) and a limit of detection (LOD) of 0.43 mM (*S*/*N* = 3) in a linear concentration range from 0.8 to 50 mM (Fig. 3). In addition, the glucose sensor did not show significant cross-reactivity to fructose, maltose or lactose. The C4-HSL sensor showed high specificity, with a sensitivity of 42 ± 1.73 µM/nM (*R*^2^ = 0.99) and an LOD of 0.12 nM in a linear range of 6.25–800 nM. A negligible response was observed to analogs such as ɣ-butyrolactone, *N*-(3-oxohexanoyl)-*L*-homoserine lactone (3-oxo-C6-HSL), *N*-butyryl-*L*-homoserine lactone (C4-HSL), and *N*-hexanoyl*-L*-homoserine lactone (C6-HSL) (Fig. 3).

The paracetamol sensor was tested in a linear concentration range between 100 and 1000 µM; the sensitivity was found to be 10.1 ± 0.27 µA/µM (*R*^2^ = 0.99), and the LOD was found to be 82 µM (Fig. 4). A negligible response was observed for caffeine, procainamide, and ethyl 4-aminobenzoate. The THC sensor response displayed a sensitivity of 7.2 ± 0.45 µA/µM (*R*^2^ = 0.98) with an LOD of 0.05 µM in a concentration range from 0.1 to 1000 µM. No cross reactivity was observed for cannabidivarin, 11-nor-9-carboxy-Δ9-tetrahydrocannabinol (THC-COOH), and caffeine (Fig. 4).

As a final example, the trypsin sensor displayed good sensitivity of 0.25 ± 0.01 µA/nM (*R*^2^ = 0.99) and an LOD of 0.20 nM in a linear range of 6.5–100 nM (Fig. 5). No cross reactivity was observed when tested with pepsin and avidin. The summarized performance of these sensors and the characteristics of the NanoMIPs are shown in Table 3.

**Table 3:** Sensor characteristics and performance

Target	Glucose^a^	C4-HSL^a^	Paracetamol^b^	THC^b^	Trypsin^b^
Sensitivity	5.6 µA/mM	42 µA/nM	10.1 µA/µM	7.2 µA/µM	0.25 µA/nM
LOD	0.4 mM	0.1 nM	82 nM	50 nM	0.2 nM
Linear range	0.8–50 mM	6.2–800 nM	100–1000 µM	0.1–1000 µM	6.5–100 nM

^a^Tested in buffer

^b^Tested in human plasma

In contrast to traditional detection methods, the present detection technology is based on electroactive e-MIPs that combine the roles of recognition elements and reporters. Conversely, traditional methods involve the monitoring of the redox activity of an analyte, which is affected by interference and the nature of the sample. The present work allows for these issues to be overcome. The sensors were designed for a single use and were quite robust. The shelf life was found to be at least 6 weeks for sensors stored at 25 ± 2 °C with relative humidity set at 60 ± 5%. Under these conditions, the sensors exhibited good recovery (93–109%).

The benefits of the present technology include the generic nature of MIPs and MIP-based sensors, the integration of biorecognition and signal generating capability within the same material (e-MIPs), the robust nature of e-MIPs and sensor devices, and an easy fabrication process that relies on well-established protocols. The fabrication process of e-MIPs involves solid-phase synthesis, which is a relatively low cost, efficient, and automated process^15,31^. The fabrication of e-MIP-based sensors here is based on well-established protocols, describing the relatively simple process of covalent attachment of nanoparticles to thiol-coated slides^19,20^. It might also be possible to deposit e-MIPs onto electrode surfaces by ink jet printing, screen printing, soft lithography, contact printing, 3D printing, and roll-to-roll processing^34^. The main technological advantage of these sensors over traditional sensors is their easy production and cost efficiency. This sensor synthesis process can be applied to manufacture low-cost disposable and portable devices designed specifically to work with microvolumes of the sample in question and that are ideal for routine work. These sensors are preferred to sensitive laboratory-based techniques in challenging applications (e.g., the prevention of terrorist activities by monitoring explosives, chemical and biological warfare agents, drugs, and toxins)^34^. e-MIP sensors can potentially be employed in clinical point-of-care diagnostics, as well as environmental, defense, and food monitoring applications.

## Materials and methods

### Synthesis of nanoparticles on the solid phase

The solid-phase synthesis of imprinted nanoparticles was carried out according to a well-established protocol^35^. Briefly, the process includes (a) activation of the solid phase (glass beads) and its silanization, (b) template immobilization, and (c) polymerization and purification of e-MIPs.

### Glass bead activation

Glass beads (60 g, 150–200 µm) were boiled in 4 M sodium hydroxide (1.2 mL of solution per g of glass beads) for 15 min and then rinsed thoroughly with deionized water (eight times, with 200 mL). Subsequently, glass beads were incubated for 60 min in a solution of 20% (v/v) sulfuric acid (1.2 mL of solution per g of glass beads). Afterward, glass beads were washed with 5 mM PBS, double-distilled water and acetone (three times, 200 mL), and then beads were finally dried in the oven at 120 °C for 15 min.

### Silanization

Activated glass beads were incubated for 8 h at 112 °C under reflux in a solution comprising 6% (v/v) silane linker and 0.24% (v/v) 1,2-bis(triethoxysilyl)ethane in toluene. Afterward, beads were washed with acetone (200 mL) and then dried for 15 min under vacuum. Finally, glass beads were cured at 120 °C for 30 min.

### Template immobilization on glass beads

Trypsin, paracetamol, and C4-HSL were immobilized on silanized glass beads using the silane derivative N-[3-(trimethoxysilyl)-propyl]-ethylenediamine. To immobilize trypsin, (20 g) silanized glass beads were incubated in a 7% (v/v) glutaraldehyde aqueous solution for 2 h. After brief washing with water (200 mL), glass beads were incubated in 25 mL of a solution of trypsin (1 mg mL^−1^) and PBS (5 mM, pH 7.2) overnight. Afterward, glass beads were washed with water (200 mL) and incubated in 25 mL of a 0.1 mM ethanolamine aqueous solution for 15 min. Subsequently, trypsin-derivatized glass beads were incubated in 25 mL of a sodium cyanoborohydride aqueous solution (1 mg mL^−1^) for 30 min. In the case of paracetamol, a carboxyl paracetamol derivative (0.5 mg mL^−1^) was immobilized onto glass beads using carbodiimide chemistry. For C4-HSL immobilization, silanized glass beads were modified with dodecanedioic acid. Subsequently, glass beads were incubated for 2 h in a 0.43 mM (S)-(−)-α-amino-γ-butyrolactone hydrobromide solution in 0.1 M MES buffer (pH 6.0). THC was immobilized on glass beads using (3-glycidyloxy-propyl)-trimethoxy-silane. For this immobilization, glass beads were incubated in a solution of THC (0.5 mg mL^−1^) and EIPA in acetonitrile (2 mg mL^−1^) at 5% (v/v) water for 24 h. To conclude, all derivatized glass beads were washed with distilled water (200 mL) then acetone (200 mL) and dried before use.

### Aqueous synthesis of e-MIPs

Aqueous solid-phase synthesis was used for the preparation of e-MIPs imprinted with trypsin. The polymerization mixture was composed of N-isopropylacrylamide (39 mg, 0.214 mmol), N,N-methylene-bis-acrylamide (6 mg, 0.078 mmol), tert-butyl acrylamide (33 mg, 0.264 mmol), acrylic acid (2.2 μL, 0.0224 mmol), ferrocenylmethyl methacrylate (FcMMA) (7 mg, 0.0281 mmol), and N-(3-aminopropyl) methacrylamide hydrochloride (NAPMA) (5.8 mg, 0.0325 mmol). The monomers were dissolved in water (100 mL), 50 mL of which was mixed with 60 g of derivatized glass beads. This mixture was sonicated for 5 min and then purged with nitrogen for 30 min.

Glucose e-MIPs were prepared by controlled precipitation polymerization by adding glucose (30 mg, 0.1665 mmol) to the polymerization mixture. Polymerization was initiated by the addition of ammonium persulfate (0.5 mL, 60 mg mL^−1^) and tetramethylethane-1,2-diamine (900 μL, 30 μL/mL) and was carried out at room temperature for 1 h. Afterward, the polymerization was stopped by adding sodium nitrite (50 mg, 0.7247 mmol), after which the polymerization solution was saturated with oxygen. The beads were then washed with water (10 bead volumes, 50 mL at 0 °C) at room temperature using a fritted (20 µm porosity) solid-phase extraction (SPE) cartridge. High-affinity e-MIPs were eluted with water at 60 °C (5 bead volumes, 20 mL). Subsequently, nanoMIPs were purified using a centrifuge cartridge filter (10 kDa).

### Organic synthesis of e-MIPs

Organic solid-phase synthesis of e-MIPs was employed for THC, paracetamol and C4-HSL. For this, the monomer mixture was prepared by mixing methacrylic acid (MAA) (1.44 g, 16.7 mmol), ethylene glycol dimethacrylate (1.62 g, 8.2 mmol), trimethylolpropane trimethacrylate (1.62 g, 4.8 mmol), N,N-diethyldithiocarbamic acid benzyl ester (0.37 g, 1.5 mmol), pentaerythritol-tetrakis-(3-mercaptopropionate) (0.09 g, 0.2 mmol), and FcMMA (0.14 g, 0.49 mmol). The components of the monomer mixture were dissolved in DMF (25 mL), and then the solution was degassed with nitrogen for 10 min. For paracetamol imprinting, instead of MAA, itaconic acid (1.7 g, 13.1 mmol) was added to the original composition. In the case of THC, N,N′-methylene-bis-acrylamide (1.29 g, 8.4 mmol), NAPMA (0.112 g, 0.6 mmol), and acrylamide (1.19 g, 16.7 mmol) were added to the original composition. The variations in the monomer mixture were introduced as a result of an optimization process performed separately. Glass beads with immobilized templates (30 g) were degassed under vacuum for 20 min and coated with the monomer mixture. Polymerization was initiated by exposing the mixture to UV light for 2 min (Philips model HB/171/A, 4 × 15 W/amps). After polymerization, the crude reaction mixture was transferred into an SPE cartridge (20 µm frit) and washed with acetonitrile at 0 °C to remove residues and side products. High-affinity e-MIPs were extracted by elution at 60 °C using a solution of 10% (v/v) ethanol in water. e-MIPs were purified using a centrifuge cartridge filter (10 kDa).

### e-MIP immobilization

Drop-sense SPGEs (DRP-250AT), with a platinum counter electrode, silver/silver chloride as a reference and dimensions of 3.4 × 1.0 × 0.05 cm (*L* × *W* × *H*), were washed with isopropanol and distilled water and then dried under a nitrogen stream. Their surface was activated by a 5 min treatment in hydrogen plasma at 20 W using a plasma barrel reactor (K1050x, Emitech, UK). e-MIPs were covalently attached to SPGEs using thioalkane linkers. For that, SPGEs were incubated in a 3 mM cysteamine ethanolic solution for 8 h. The SPGE surface was rinsed with ethanol and incubated for 30 min in a 100 µL solution comprising 0.03 mg mL^−1^ e-MIPs, 0.4 M EDC, and 0.1 M NHS in 5 mM PBS and rinsed with ultrapure water.

### Electrochemical measurements

All experiments were carried out using an Autolab11 instrument (Netherlands). DVP was recorded in the potential range from −0.4 to 0.8 V, at a scan rate of 20 mV/s and a step potential and modulation amplitude of 50 mV. The current signal was measured at the redox potential of the redox marker (FcMMA) in e-MIPs (0.22 V vs Ag/AgCl).

## References

[1] Ugalmugale, S., Swain, R. & (Global Market insights Inc., 2018).

[2] Sadana, A., Sadana, N. & Sadana, R. *A Fractal Analysis of Chemical Kinetics with Applications to Biological and Biosensor Interfaces*. 1st ed., p. 337 (Elsevier Science, 2018).

[3] Berti F (2010). Quasi-monodimensional polyaniline nanostructures for enhanced molecularly imprinted polymer-based sensing. Biosens. Bioelectron..

[4] Korposh S (2014). Selective vancomycin detection using optical fibre long period gratings functionalised with molecularly imprinted polymer nanoparticles. Analyst.

[5] Altintas Z (2015). Detection of waterborne viruses using high affinity molecularly imprinted polymers. Anal. Chem..

[6] Smolinska-Kempisty K (2017). New potentiometric sensor based on molecularly imprinted nanoparticles for cocaine detection. Biosens. Bioelectron..

[7] Canfarotta F (2018). A novel capacitive sensor based on molecularly imprinted nanoparticles as recognition elements. Biosens. Bioelectron..

[8] Sergeyeva T (2019). Development of a smartphone-based biomimetic sensor for aflatoxin B1 detection using molecularly imprinted polymer membranes. Talanta.

[9] Chen L, Wang X, Lu W, Wu X, Li J (2016). Molecular imprinting: perspectives and applications. Chem. Soc. Rev..

[10] Esen C, Czulak J, Cowen T, Piletska E, Piletsky SA (2018). Highly efficient abiotic assay formats for methyl parathion: molecularly imprinted polymer nanoparticle assay as an alternative to enzyme-linked immunosorbent assay. Anal. Chem..

[11] Chen L, Muhammad T, Yakup B, Piletsky SA (2017). New immobilisation protocol for the template used in solid-phase synthesis of MIP nanoparticles. Appl. Surf. Sci..

[12] Rangel PXM (2019). Solid-phase synthesis of molecularly imprinted polymer nanolabels: affinity tools for cellular bioimaging of glycans. Sci. Rep..

[13] Garcia-Cruz A, Cowen T, Voorhaar A, Piletska E, Piletsky SA (2020). Molecularly imprinted nanoparticles-based assay (MINA)-detection of leukotrienes and insulin. The Analyst.

[14] Poma A, Guerreiro A, Caygill S, Moczko E, Piletsky S (2014). Automatic reactor for solid-phase synthesis of molecularly imprinted polymeric nanoparticles (MIP NPs) in water. RSC Adv..

[15] Poma A (2013). Solid‐phase synthesis of molecularly imprinted polymer nanoparticles with a reusable template–“plastic antibodies”. Adv. Funct. Mater..

[16] Mazzotta E, Picca R, Malitesta C, Piletsky S, Piletska E (2008). Development of a sensor prepared by entrapment of MIP particles in electrosynthesised polymer films for electrochemical detection of ephedrine. Biosens. Bioelectron..

[17] Prasad K, Prathish K, Gladis JM, Naidu G, Rao TP (2007). Molecularly imprinted polymer (biomimetic) based potentiometric sensor for atrazine. Sens. Actuators B: Chem..

[18] Mazzotta E (2016). Solid-phase synthesis of electroactive nanoparticles of molecularly imprinted polymers. A novel platform for indirect electrochemical sensing applications. Sens. Actuators B: Chem..

[19] Garcia-Mutio D (2016). Solid-phase synthesis of imprinted nanoparticles grafted on gold substrates for voltammetric sensing of 4-ethylphenol. Sens. Actuators B: Chem..

[20] Garcia-Mutioa, D. et al. Molecularly imprinted high affinity nanoparticles for 4-ethylphenol sensing. (2015).

[21] Uzun L, Turner AP (2016). Molecularly-imprinted polymer sensors: realising their potential. Biosens. Bioelectron..

[22] Zhang X (2018). Electrosynthesized MIPs for transferrin: plastibodies or nano-filters?. Biosens. Bioelectron..

[23] Sharma PS, Garcia-Cruz A, Cieplak M, Noworyta KR, Kutner W (2019). “Gate effect” in molecularly imprinted polymers: the current state of understanding. Curr. Opin. Electrochem.

[24] Sergeyeva TA, Piletsky SA, Brovko AA, Slinchenko EA, Sergeeva LM, El’Skaya AV (1999). Selective recognition of atrazine by molecularly imprinted polymer membranes. Development of conductometric sensor for herbicides detection. Analytica Chim. Acta.

[25] Yoshimi Y (2019). Size of heparin-imprinted nanoparticles reflects the matched interactions with the target molecule. Sensors.

[26] Piletsky SA (1998). Imprinted membranes for sensor technology: opposite behavior of covalently and noncovalently imprinted membranes. Macromolecules.

[27] Iacob B-C, Bodoki E, Farcau C, Barbu-Tudoran L, Oprean R (2016). Study of the molecular recognition mechanism of an ultrathin MIP film-based chiral electrochemical sensor. Electrochim. Acta.

[28] Udomsap D, Branger C, Culioli G, Dollet P, Brisset H (2014). A versatile electrochemical sensing receptor based on a molecularly imprinted polymer. Chem. Commun..

[29] Saleem M (2015). Review on synthesis of ferrocene-based redox polymers and derivatives and their application in glucose sensing. Anal Chim. Acta.

[30] Piletsky S (1995). Atrazine sensing by molecularly imprinted membranes. Biosens. Bioelectron..

[31] Muzyka K, Karim K, Guerreiro A, Poma A, Piletsky S (2014). Optimisation of the synthesis of vancomycin-selective molecularly imprinted polymer nanoparticles using automatic photoreactor. Nanoscale Res. Lett..

[32] Kamra T (2016). Covalent immobilization of molecularly imprinted polymer nanoparticles on a gold surface using carbodiimide coupling for chemical sensing. J. Colloid Interface Sci..

[33] BelBruno JJ (2018). Molecularly imprinted polymers. Chem. Rev..

[34] Ahmad OS, Bedwell TS, Esen C, Garcia-Cruz A, Piletsky SA (2018). Molecularly imprinted polymers in electrochemical and optical sensors. Trends Biotechnol..

[35] López‐Puertollano D (2019). Study of epitope imprinting for small templates: preparation of nanoMIPs for ochratoxin A. ChemNanoMat.

